# Human Fetal Bone Marrow-Derived Mesenchymal Stem Cells Promote the Proliferation and Differentiation of Pancreatic Progenitor Cells and the Engraftment Function of Islet-Like Cell Clusters

**DOI:** 10.3390/ijms20174083

**Published:** 2019-08-21

**Authors:** Xing Yu Li, Shang Ying Wu, Po Sing Leung

**Affiliations:** School of Biomedical Sciences, Faculty of Medicine, The Chinese University of Hong Kong, Shatin, Hong Kong, China

**Keywords:** diabetes, apoptosis, PI3K, MAPK, co-culture, islet-like cell clusters, co-transplantation

## Abstract

Pancreatic progenitor cells (PPCs) are the primary source for all pancreatic cells, including beta-cells, and thus the proliferation and differentiation of PPCs into islet-like cell clusters (ICCs) opens an avenue to providing transplantable islets for diabetic patients. Meanwhile, mesenchymal stem cells (MSCs) can enhance the development and function of different cell types of interest, but their role on PPCs remains unknown. We aimed to explore the mechanism-of-action whereby MSCs induce the in vitro and in vivo PPC/ICC development by means of our established co-culture system of human PPCs with human fetal bone marrow-derived MSCs. We examined the effect of MSC-conditioned medium on PPC proliferation and survival. Meanwhile, we studied the effect of MSC co-culture enhanced PPC/ICC function in vitro and in vivo co-/transplantation. Furthermore, we identified IGF1 as a critical factor responsible for the MSC effects on PPC differentiation and proliferation via IGF1-PI3K/Akt and IGF1-MEK/ERK1/2, respectively. In conclusion, our data indicate that MSCs stimulated the differentiation and proliferation of human PPCs via IGF1 signaling, and more importantly, promoted the in vivo engraftment function of ICCs. Taken together, our protocol may provide a mechanism-driven basis for the proliferation and differentiation of PPCs into clinically transplantable islets.

## 1. Introduction

Diabetes mellitus (DM) is an endocrine disease with chronic complications, which are primarily characterized with insulin-deficiency because of pancreatic beta-cell loss. Patients with type 1 diabetes mellitus (T1DM) and severe cases of type 2 diabetes mellitus (T2DM) require daily injection of insulin [1,2]. However, life-long insulin injection leads to complicated side effects, notably hypoglycemia. In view of this fact, patients need to regain their physiological insulin secretion in response to glucose rather than using insulin therapy alone [3]. Islet transplantation is a curable option for the patients with insulin-dependent diabetes mellitus. Owing to the inherent lack of islet donors, exploring the replacement of islets gives rise to the boom of pancreatic stem cell research. Pancreatic stem cells are the source of all pancreatic cells and can differentiate into insulin-producing islet-like cell clusters, without processing complicated stages of embryonic development [4]. In light of this, enhancement for the proliferation and differentiation of pancreatic progenitor cells (PPCs) provides numerous sources and potential alternatives of islets.

Mesenchymal stem cells (MSCs) play a critical role in supporting many different stem cells development in vivo [5], while the in vitro cultures were initially done by scientists in the late 1960s, which were characterized by small body and relatively long cell processes [6]. They can be isolated from a variety of sources of bone marrow to human organs [7,8]. These multipotent cells possess both differentiation and self-renewal ability, but unlike other stem cells, MSCs are more genetically stable and survive longer in the in vitro setting [9]. Besides, the immunomodulatory effects of MSCs make MSCs ideal for stem cell therapy in many diseases [10,11,12,13,14]. To achieve an in vitro microenvironment, scientists have also attempted to introduce MSCs into the culture system. By doing so, co-culture with MSCs is able to promote the proliferation and differentiation, as demonstrated by cardiac stem cells [15]. However, the effects of MSC co-culture on the development of human PPCs/islet-like cell clusters (ICCs) are largely ambiguous.

Insulin-like growth factor 1 (IGF1) is primarily expressed by the liver and participates in cell growth, survival, and differentiation [16,17,18,19]. IGF1 is a crucial morphogenic factor secreted from MSCs known to be responsible for the wound healing effect of MSCs [20]. Prior studies revealed that IGF1 and IGF1 receptor (IGF1R) play pivotal roles in not only embryonic development but also postnatal growth, and some of the IGF1R mutations even turned out to be lethal in mice [21]. To our knowledge, IGF1 mediates the proliferation and development of pancreatic β-cells [22,23,24]. It has been previously shown that the increase of IGF1 in beta-cell local expression can stimulate islet regeneration and alleviate T1DM in mice [25]. However, the exact role of IGF1 in the proliferation and differentiation of human PPCs has yet to be further investigated.

Against this background, we aimed to explore the mechanism-of-action whereby MSCs induce PPC proliferation and differentiation by means of our established co-culture system of human PPCs with human fetal bone marrow-derived MSCs. To address this issue, we examined the levels of proliferation and cell death of PPCs treated with MSC-conditioned medium as well as employed an IGF1R inhibitor—picropodophyllin (PPP)—to verify the proposed pathways of IGF1-PI3K/Akt and MEK/ERK1/2 involved. We thus established co-culture system of MSCs-PPCs/ICCs both in vitro and in vivo to induce the differentiation of PPCs into ICCs and to conduct co-transplantation. Our results revealed that MSCs could promote the proliferation and differentiation of PPCs and the post-transplantation function of ICCs.

## 2. Results

### 2.1. Human Fetal Bone Marrow-Derived MSCs Enhances PPC Proliferation

**Figure 1:**
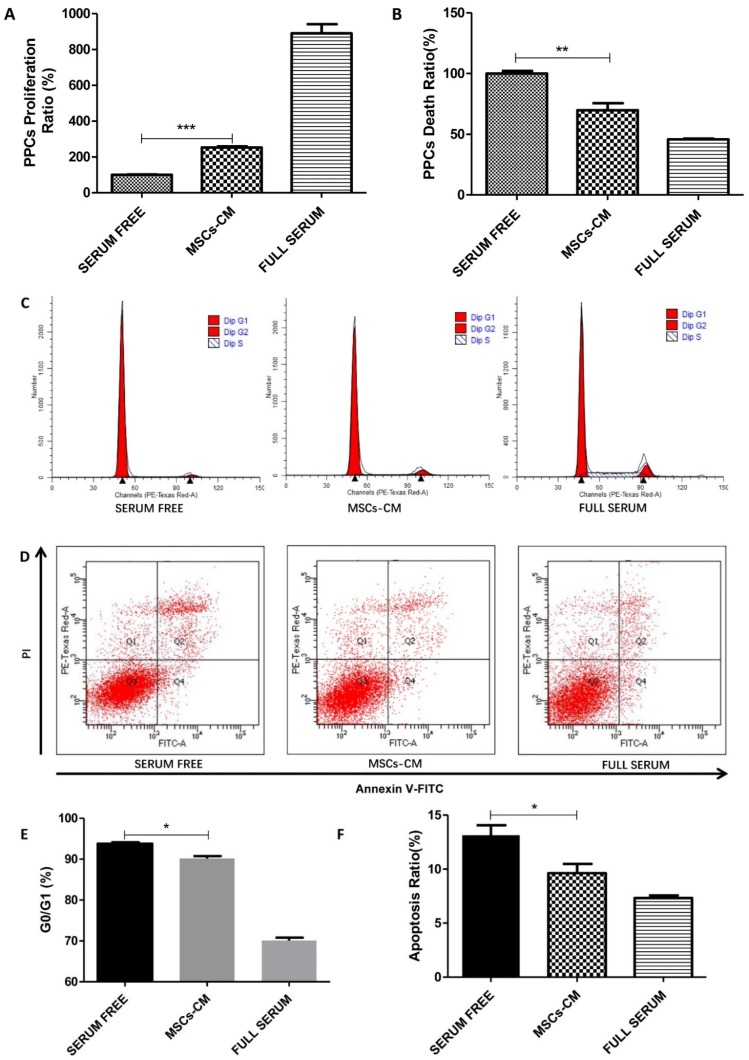
MSC-conditioned medium (MSCs-CM) mediated induction of cell proliferation and cell death in human pancreatic progenitor cells (PPCs). (**A**) PPCs were cultured in MSCs-CM or serum-free medium for 48 hours. A BrdU assay was performed to measure the proliferation of PPCs. (**B**) PPCs were cultured in conditions of serum-free, MSCs-CM, or full serum medium for 48 hours. A cell death assay was conducted to assess cell death. (**C**,**E**) Cell cycle distribution was measured by flow cytometry. (**D**,**F**) An Annexin V assay was also conducted to measure the apoptotic rate of PPCs. Q1 represents dead cells; Q2 and Q4 represent early and late apoptosis, respectively; Q3 represents non-apoptotic cells. (n = 3~5 per group; * *p* < 0.05, ** *p* < 0.01, *** *p* < 0.001. All data are expressed as means ± SEM).

To study the effects of MSCs on PPC growth, PPCs were cultured in serum-free or MSC-conditioned medium (MSCs-CM) for 48 h prior examination by the BrdU assay. Our results showed that MSCs-CM increased the levels of PPC proliferation when compared with those of the starvation group, as shown in Figure 1A. Meanwhile, we found that the starvation condition that induced cell death could be rescued by MSCs-CM treatment, as demonstrated by a cell death ELISA kit, as shown in Figure 1B. Consistently, results from cell cycle distribution of PPCs also revealed starvation induced cell growth arrested in the G0/G1 phase, and these demonstrated effects being blocked by MSCs-CM, as shown in Figure 1C,E. Moreover, these observations were further confirmed by data from an Annexin V assay, showing that MSCs-CM was able to decrease the apoptosis rate in PPCs, as shown in Figure 1D,F.

**Figure 2:**
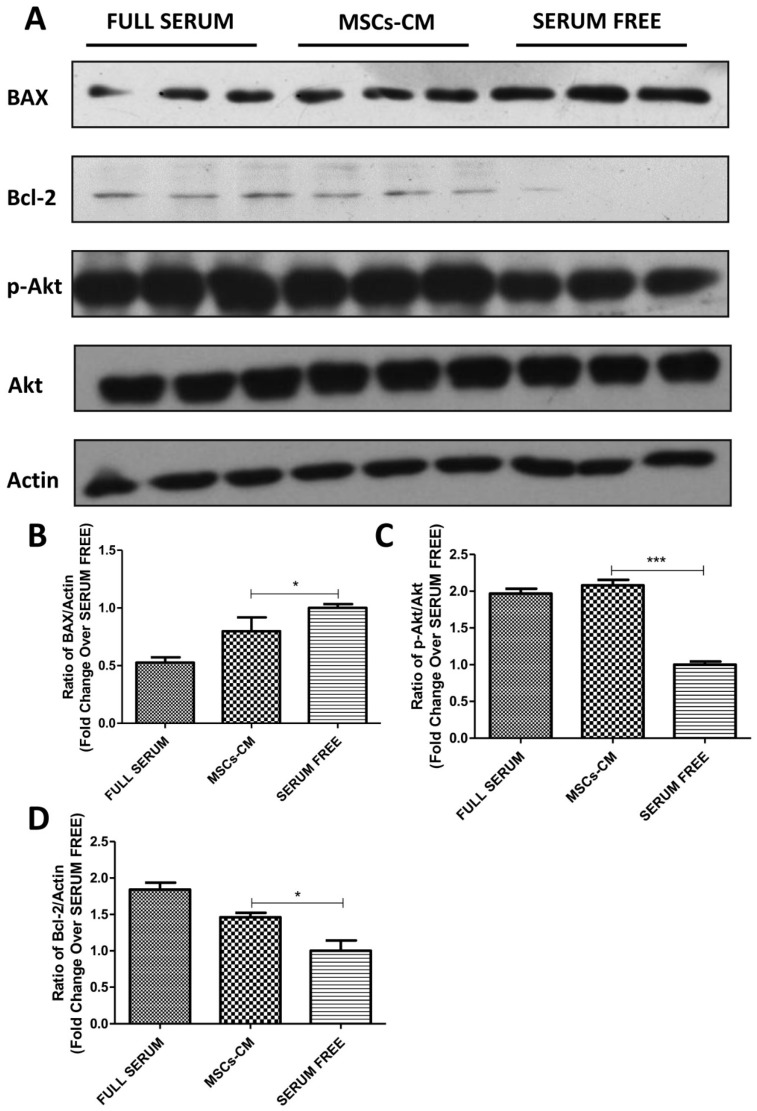
MSCs-CM mediated amelioration of human PPC apoptosis induced by starvation. PPCs were cultured under the conditions of serum-free, MSCs-CM, or normal full serum for 48 h. (**A**) Western blot analyses of Bcl-2, BAX, and Akt phosphorylation levels were examined and (**B**,*C*,*D*) quantified by ImageJ software. (n = 3 per group; * *p* < 0.05, ** *p* < 0.01, *** *p* < 0.001. All data are expressed as means ± SEM).

To further study the MSCs-CM induced PPC proliferation and apoptosis, we measured the protein expression of anti-apoptotic molecule B-cell lymphoma 2 (Bcl-2), Bcl-2-associated X protein (apoptosis regulator BAX), and Akt. Western blot analyses showed that Bcl-2 was significantly up-regulated under MSCs-CM culture relative to the serum-free condition. The expression of BAX was up-regulated after starvation; however, the MSCs-CM condition decreased the BAX expression level, indicating that MSCs-CM ameliorated the apoptosis induced by starvation, which were further confirmed by up-regulated levels of phosphorylated Akt under the MSCs-CM condition, as shown in Figure 2A–D.

### 2.2. IGF1 is Involved in MSC-Induced PPC Proliferation

**Figure 3:**
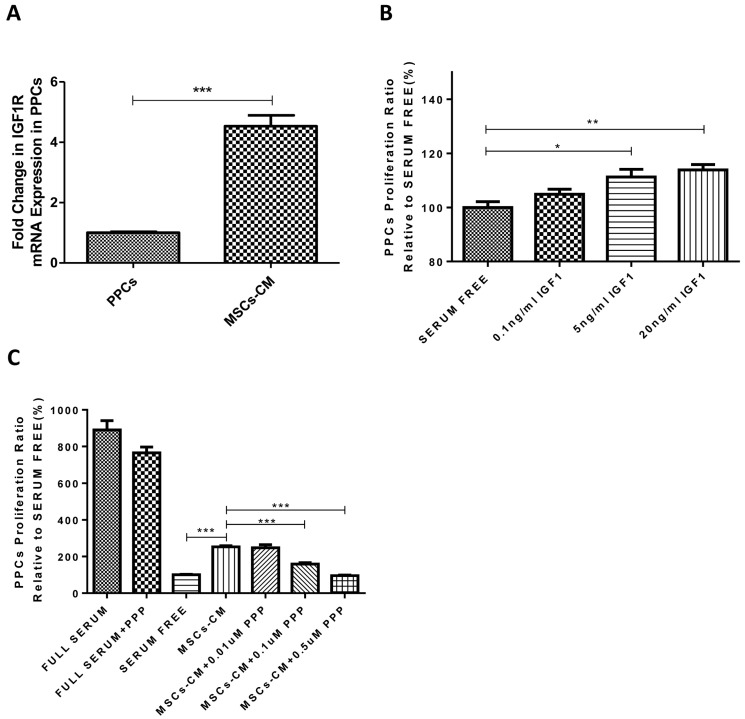

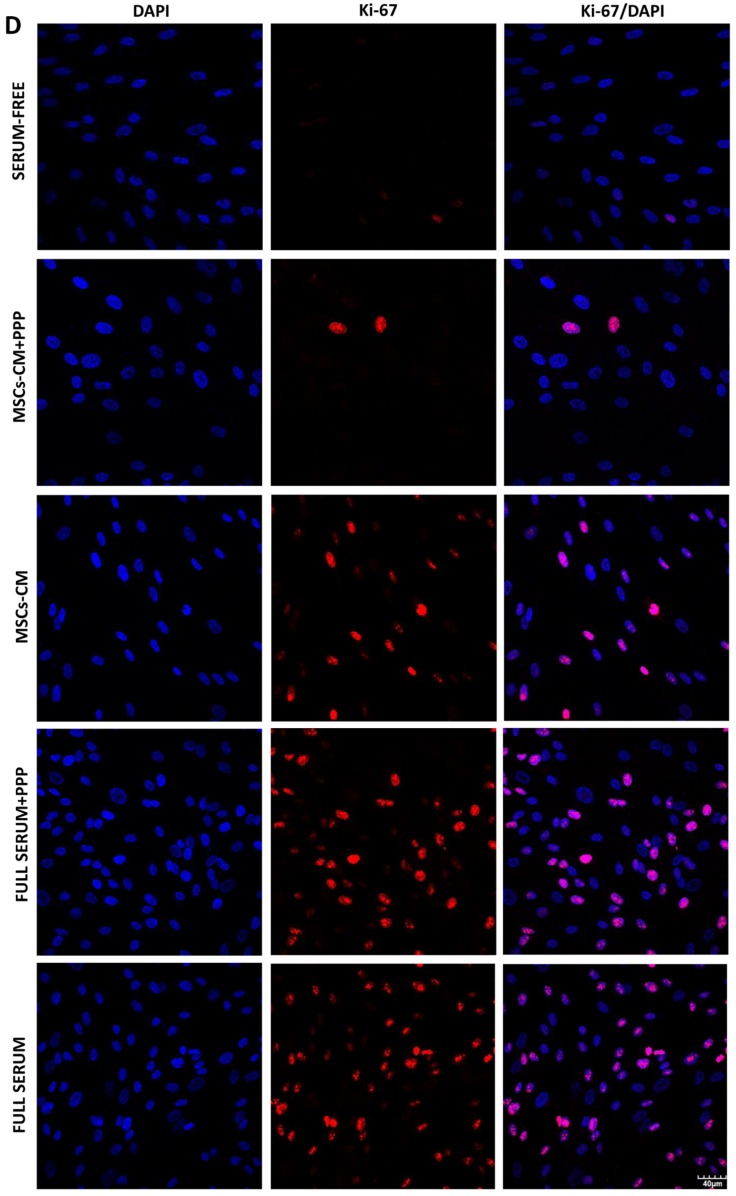

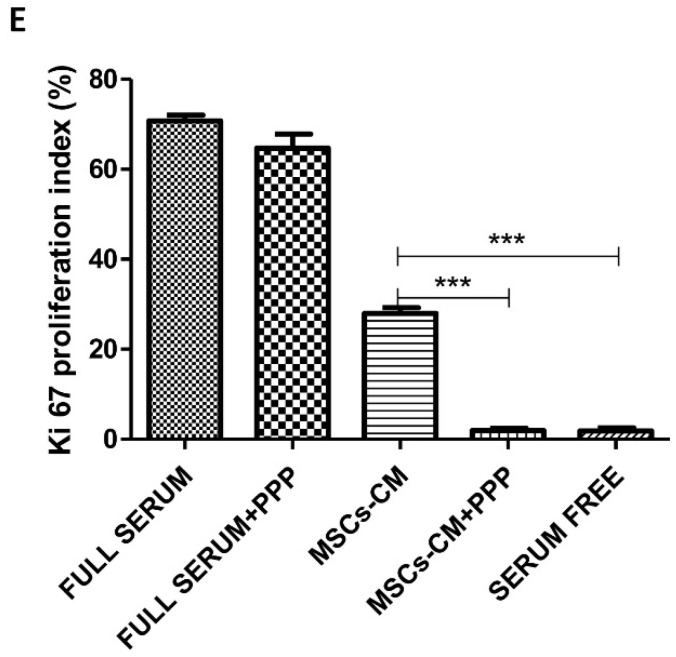
Regulatory role of IGF1 in the determination of MSC-induced PPC proliferation. (**A**) PPCs were cultured with MSCs-CM for 48 h and processed the analysis of IGF1R gene expression. (**B**) A BrdU assay was performed to evaluate the proliferation induced by additional IGF1 at the dosage of 0.1, 5, and 20 ng/ml. (**C**) MSCs-CM induced PPC proliferation was detected by BrdU assay with the administration of picropodophyllin (PPP) in a dose dependent manner (0.01, 0.1, and 0.5 μM). PPCs were cultured in full serum, MSCs-CM, or serum-free conditions in the presence or absence of PPP (an IGF1R inhibitor) for 48 h prior to immunofluorescent analysis of Ki67 (**D**) and (**E**). DAPI and Ki67 staining were expressed in blue and red, respectively. Scale bar = 40 μm. (n = 5 per group; * *p* < 0.05, ** *p* < 0.01, *** *p* < 0.001. All data are expressed as means ± SEM).

As we observed, the gene expression level of IGF1R in PPCs was increased under the MSCs-CM condition (4.53-fold, p < 0.001), in relation to the normal condition, as shown in Figure 3A, indicative of a potential role of IGF1 in the MSCs-PPCs culture system. To proceed to the role of IGF1 on PPCs, we examined the PPC proliferation rate with exogenous administration of IGF1. By means of BrdU experiments, we found that IGF1 promoted PPC growth in a dose dependent manner (0.1, 5, and 20 ng/ml), as shown in Figure 3B. Moreover, we also identified IGF1 being a key factor in MSC-induced PPC proliferation, of which the effect was diminished by the application of PPP, an IGF1R inhibitor. We found that MSC-induced PPC proliferation was decreased by PPP in a dose dependent manner (0.01, 0.1, and 0.5 μM), as demonstrated by a BrdU assay, as shown in Figure 3C. Furthermore, immunofluorescent staining of Ki-67 confirmed administration of PPP (0.5 µM) being able to reduce the Ki-67 positive cells, thus subsequently inhibiting the action of MSCs-CM in PPC proliferation, as shown in Figure 3D,E.

### 2.3. IGF1 Activates Akt and ERK in MSC-Induced PPC Proliferation

**Figure 4:**
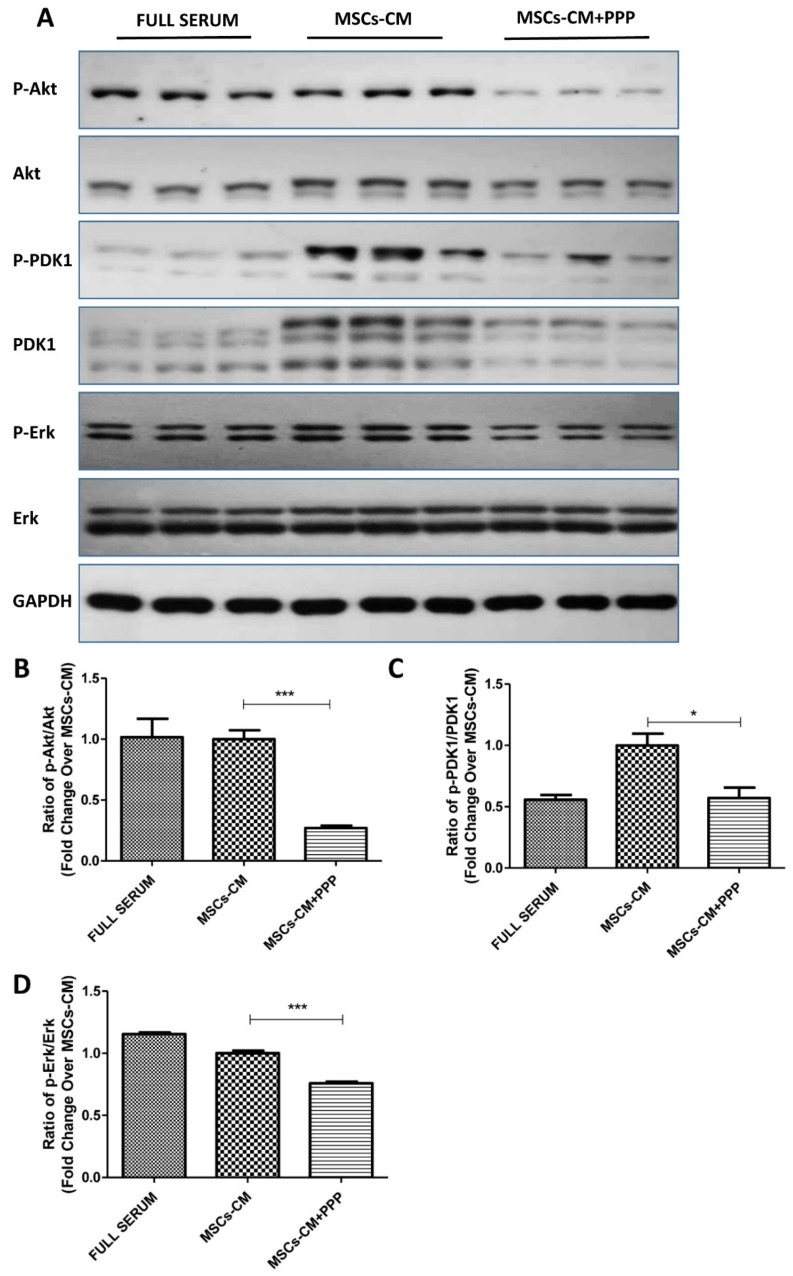
Western blot analysis of IGF1-mediated downstream signaling pathways. (**A**) PPCs were cultured under the conditions of full serum and MSCs-CM with or without PPP for 48 h and harvested for analyses of the phosphorylation of Akt, PDK1, and ERK1/2. (**B**–**D**) Quantification was conducted using ImageJ software. (n = 3 per group; * *p* < 0.05, ** *p* < 0.01, *** *p* < 0.001. All data are expressed as means ± SEM).

We then sought to examine the downstream pathways of IGF1 involved in the presence or absence of PPP (0.5 µM) in MSCs-CM. As shown by western blot results, PPP inhibited the phosphorylation of Akt, PDK1, and ERK1/2, as shown in Figure 4A–D, under the MSCs-CM condition, suggesting the involvement of PI3K/Akt and MEK/ERK1/2 pathways. 

### 2.4. Human Fetal Bone Marrow-Derived MSCs Promote PPC Differentiation into ICCs

**Figure 5:**
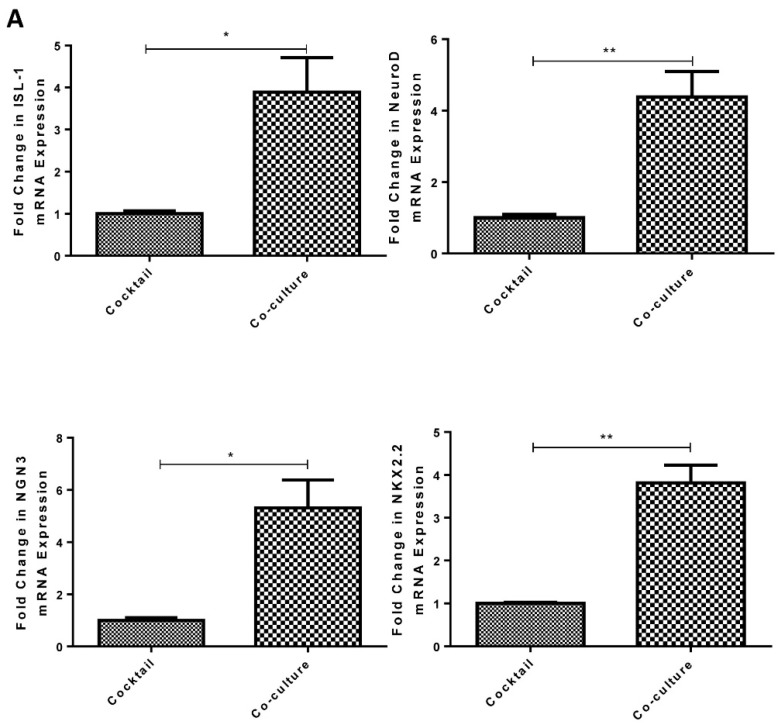

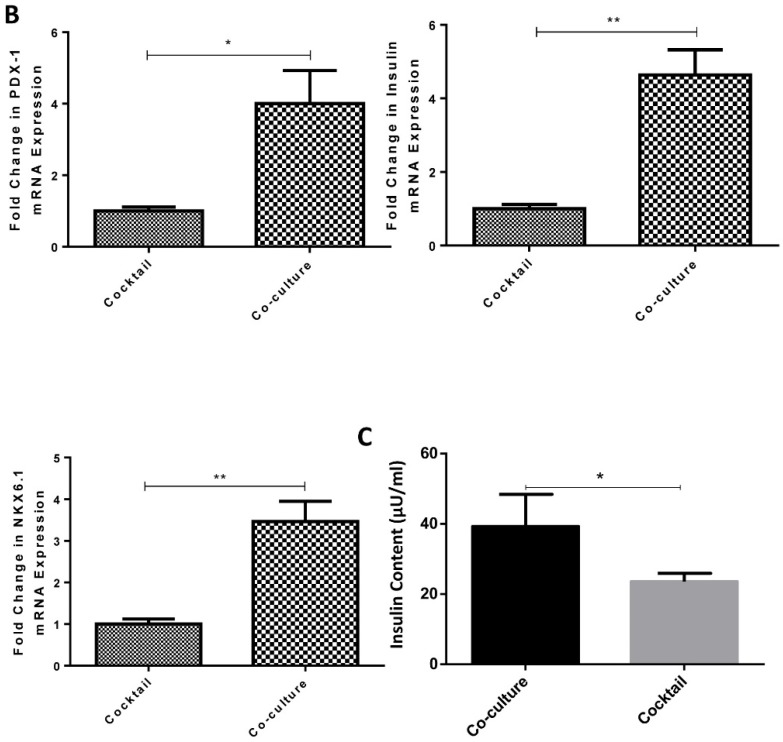
Promotion of the differentiation of human PPCs into islet-like cell clusters (ICCs) under the MSC co-culture condition. (**A**) Gene expression of MSC co-culture derived ICC differentiation markers (NGN3, NKX2.2, and NeuroD, ISL-1), and (**B**) maturation markers (PDX1, insulin, and NKX6.1) markers were measured by real-time PCR in relation with the differentiation cocktail-derived ICCs. (**C**) Insulin content of ICCs was performed by an ultra-sensitive insulin ELISA kit. (n = 3 per group; * *p* < 0.05, ** *p* < 0.01, *** *p* < 0.001. All data are expressed as means ± SEM).

The functionality of the resultant ICCs was evaluated by comparison of the differentiation and maturation markers between co-culture and cocktail-derived ICCs using real-time PCR assay. Human MSC co-culture derived ICCs were found to express higher levels of differentiation markers, such as ISL-1 (3.39-fold, P < 0.05), NeuroD (4.38-fold, P < 0.01), NKX2.2 (3.82-fold, P < 0.01), and NGN3 (5.31-fold, P < 0.05), as shown in Figure 5A; more importantly, the maturation markers, e.g., PDX1 (4.01-fold, P < 0.05), insulin (4.64-fold, p < 0.01), as shown in Figure 5B, and NKX6.1 (3.47-fold, P < 0.01), were also higher expressed when compared with those of the cocktail-derived ICCs. In addition, co-culture derived ICCs had higher insulin content than the cocktail control, as shown in Figure 5C.

### 2.5. IGF1 is Involved in MSC Co-Culture Induced PPC Differentiation

**Figure 6:**
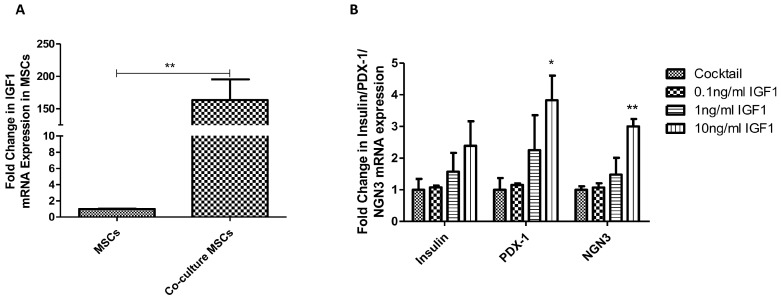

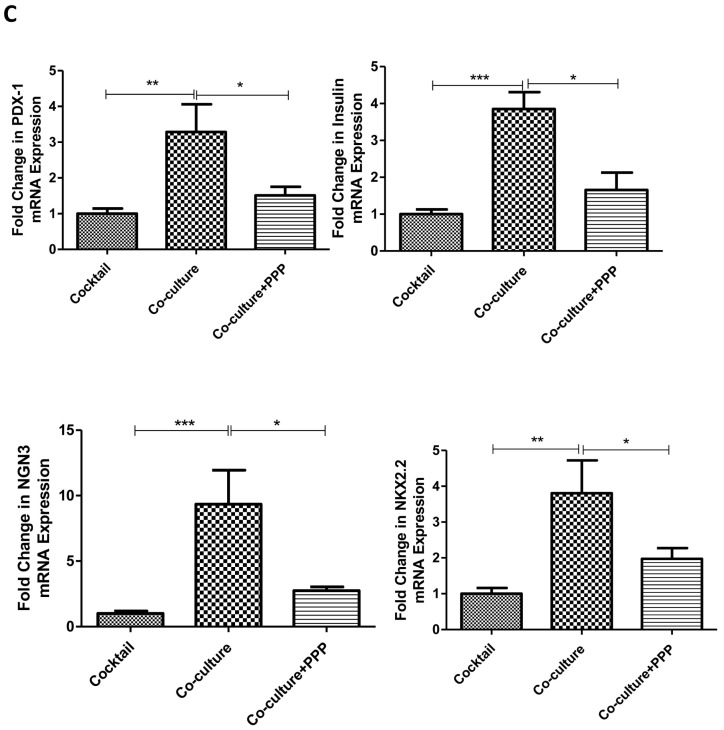
Involvement of IGF1 in the MSC-mediated PPC differentiation into ICCs. (**A**) IGF1 gene expression was elevated after co-culture as demonstrated by real-time PCR. The effects of IGF1 (**B**) and IGF1R (**C**) on the differentiation of PPCs were demonstrated by the expression profile of differentiation and maturation markers using real-time PCR. (n = 3 per group; * *p* < 0.05, ** *p* < 0.01, *** *p* < 0.001. All data are expressed as means ± SEM).

The gene expression level of IGF1 was significantly increased after co-culture indicating the involvement of IGF1 in co-culture induced differentiation, as shown in Figure 6A. To further study the role of IGF1 in PPC differentiation, we firstly applied IGF1 at the dosage of 0.1, 1, and 10 ng/ml into the PPCs-ICCs differentiation system. As expected, there were increased gene expression levels of ICC differentiation and maturation markers as shown in Figure 6B. Secondly, PPP was used to assess the participation of IGF1R in MSC-induced PPC differentiation. To avoid the inhibitory effect of PPP on MSCs, PPP (0.5 µM) was added into co-culture conditioned medium, which was able to partly diminish the co-culture conditioned medium-derived ICC differentiation, as evidenced by the gene expression of several differentiation and maturation markers as shown in Figure 6C.

### 2.6. PPC-Conditioned Medium Enhances Human Fetal Bone Marrow-Derived MSC Angiogenic Factor Expression

**Figure 7:**
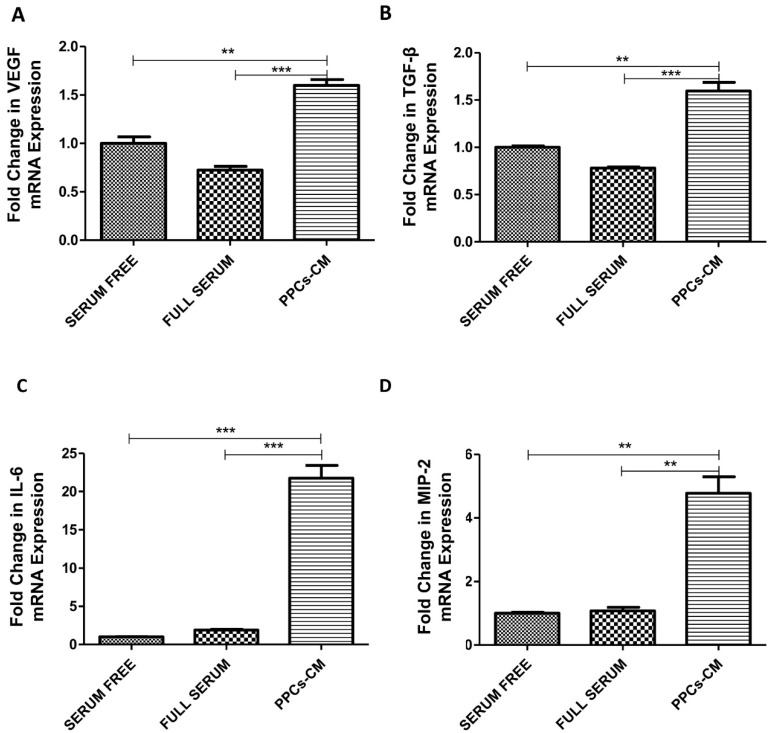
PPC-mediated increase in the expression of angiogenic factors of MSCs. (**A**–**C**) Real-time PCR was performed to measure the mRNA level of angiogenic factors VEGF, TGF-β, and IL-6 under the PPCs-CM condition or serum-free condition. (n = 3 per group; * *p* < 0.05, ** *p* < 0.01, *** *p* < 0.001. All data are expressed as means ± SEM).

We next sought to explore the potential effect on the expression of MSC angiogenic factors in exposure to the conditioned medium (PPCs-CM). Interestingly, our results showed that the gene expression levels of angiogenic factors, including VEGF (3.15-fold, p < 0.001), as shown in Figure 7A, TGF-beta (1.51-fold, p < 0.01), as shown in Figure 7B, IL-6 (10.26-fold, p < 0.001), as shown in Figure 7C, and MIP-2 (4.77-fold, p < 0.01), as shown in Figure 7D, were consistently elevated. 

### 2.7. MSCs Co-Transplantation Enhances the Engrafted Function of ICCs

**Figure 8:**
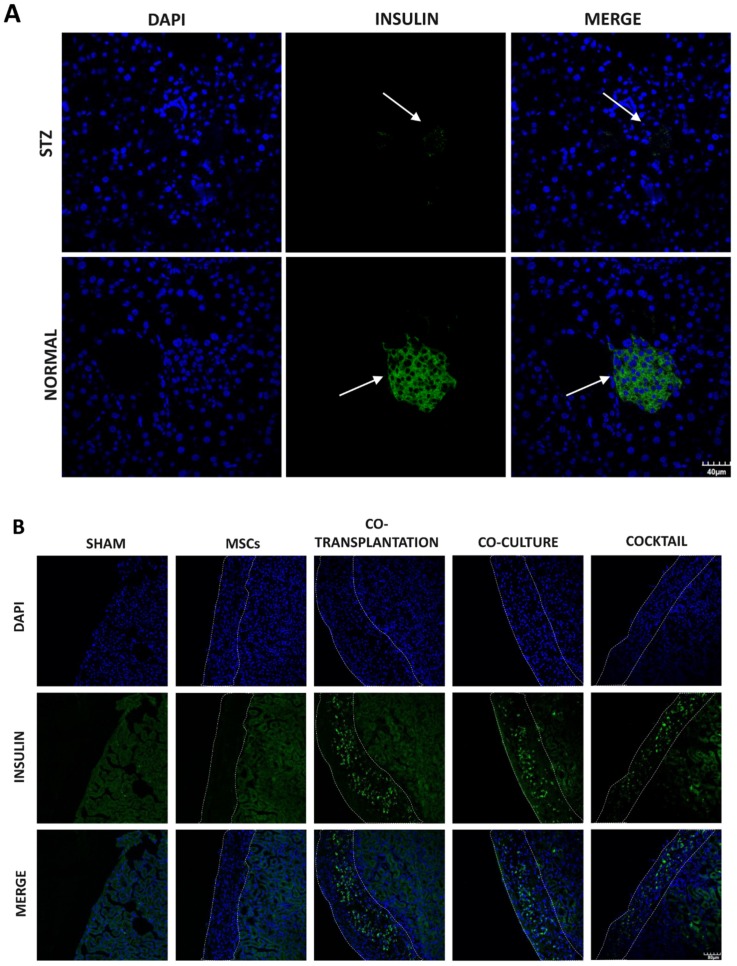

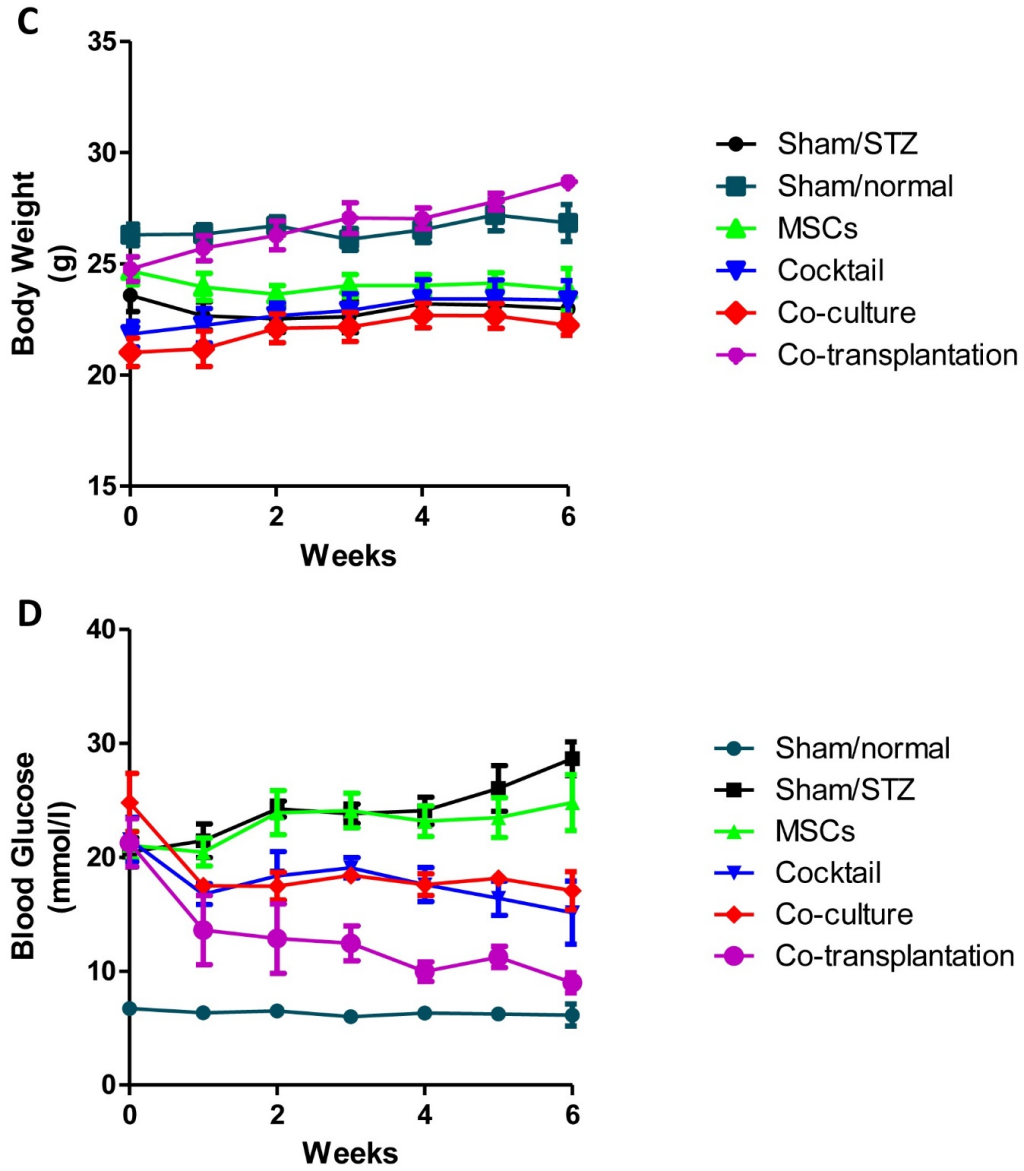

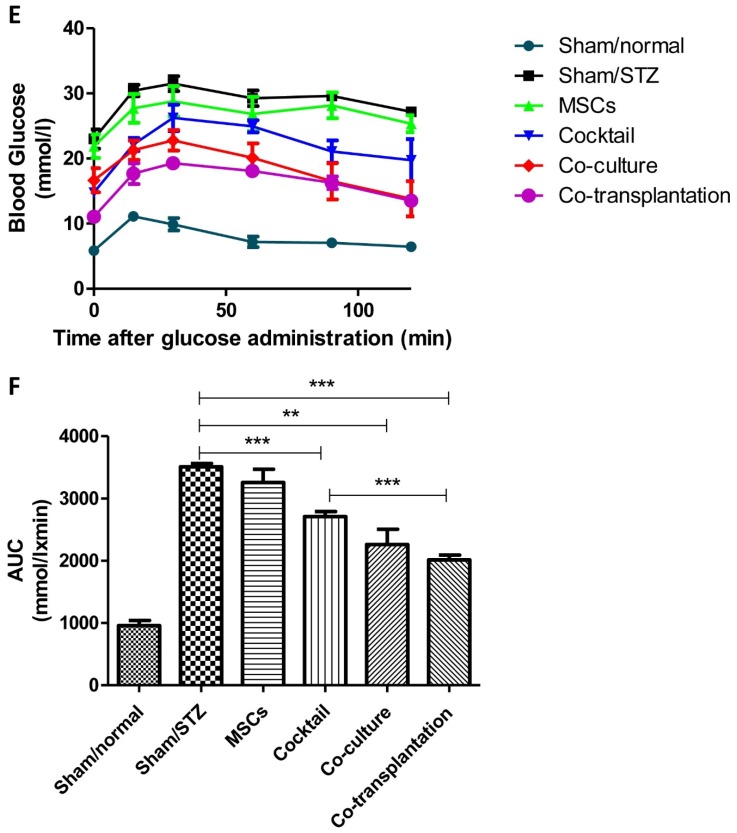
Promotion of in vivo functionality in ICC engraftment by MSC co-transplantation. (**A**) Assessment of insulin-positive pancreatic islets indicate the destruction of beta cells in nude mice with streptozocin (STZ) treatment 10 days post-transplantation. (**B**) Dashed areas represent the insulin-producing ICC engraftment in the kidney 10 days post-transplantation. The body weight (**C**) and blood glucose (**D**) were monitored every week. An intraperitoneal glucose tolerance test (IPGTT) (glucose, 1 mg/kg) (**E**) and AUCs (**F**) were conducted 1 month after transplantation. Insulin and DAPI staining were expressed in green and blue, respectively. Scale bar = 40μm. (n = 5 per group; * *p* < 0.05, ** *p* < 0.01, *** *p* < 0.001. All data are expressed as means ± SEM).

Transplantation was performed to assess the in vivo functionality of the cocktail-derived and co-culture derived ICCs; besides, ICCs were also co-transplanted with MCSs to further investigate the supporting role of MSCs in the in vivo condition. To address this issue, streptozocin (STZ)-treated nude mice with destructed β-cell mass/function were employed as the T1DM animal model, as shown in Figure 8A. The cocktail and co-culture induced ICCs as well as MSCs and MSCs/cocktail-induced ICCs were either transplanted or co-transplanted under the renal capsules of STZ-treated diabetic nude mice, as shown in Figure 8B. Post-transplantation functions were subsequently examined and compared, as demonstrated by the body weight, as shown in Figure 8C, blood glucose level, as shown in Figure 8D, and an intraperitoneal glucose tolerance test (IPGTT), as shown in Figure 8E,F.

## 3. Discussion

It has been recognized that the mesenchyme is critical for in vivo pancreas formation [26]. Recent reports have shown that rat mesenchyme acts on both upstream and downstream of *Ngn3* to direct the lineage of pancreatic β-cell fate [27]. In light of this knowledge, we aimed to utilize the niche of human MSCs, mimicking an in vitro microenvironment to promote the development of human PPCs into ICCs with transplantation potential. Our results were unambiguous in showing that co-culture with MSCs could enhance PPC proliferation as well as protect PPCs from apoptosis and cell death. It is crucial to enhance the proliferation and anti-apoptotic capacity, particularly when the PPCs/ICCs are very susceptible to in vivo hypoxia, lipotoxicity, and other stress conditions during islet transplantation in diabetic patients [28,29]. It is noteworthy that pre-treatment of MSCs-CM enhanced the differentiation ability of PPCs prior to exposure to differentiation cocktail protocol (data not shown); this observation is consistent with our findings of MSC co-culture that enhanced PPC differentiation. 

In comparison with other pluripotent stem cells, such as embryonic stem cells (ESCs), our PPCs are more unipotent and direct in differentiating into insulin-producing beta cells, thus minimizing the risks of teratoma formation that may occur during ESC therapy [30]. However, in our previous established PPC expansion and PPCs-ICCs differentiation protocol, growth factor cocktails were employed, which were not very cost effective, and the differentiation level was restricted. In recent studies, co-culture is gradually developed as a simple but effective way to enhance cell survival as well as differentiation [31]. Direct/indirect cell–cell interactions and a conditioned medium environment provide long lived and even short lived molecules secreted from one type of cells for another, supporting cell development [32]. To illustrate, the ex vivo co-culture of cord-blood with MSC improved cord-blood function followed with better performance after transplantation [33]. We thus attempted to elicit in vitro direct differentiation using human MSC co-culture with PPCs under various cell ratios (MSCs vs. PPCs) and no exogenous growth factors, finally optimizing the ratio of 1:2 by means of assessing the expression profile for the differentiation and maturation markers of our ICCs (data not shown). Based on this co-culture protocol, we observed the resultant ICCs with increases in the expression of differentiation and maturation markers as well as insulin content. However, these resultant ICCs from direct differentiation protocol were still lacking the expression of GLUT-2, which was only functionally present during post-transplantation, as we reported previously in our laboratory [4]. This sort of inherent issue still remained to be resolved in our co-culture system. As we have already known, in MSC transplantation alone exists some limitations, for example, how to trigger the in vivo directed MSC differentiation and how to maintain the engraftment [34]. As indicated in our results, the transplantation of MSCs alone failed to alleviate the hyperglycemia in T1DM animal models, suggesting that the development from MSCs into insulin-producing cells requires specific triggering factors. In view of this fact, MSCs usually function as helper cells during islet co-transplantation in order to ameliorate complications of diabetes and enhance engrafted islet function [35,36,37,38,39,40]. Interestingly, the use of MSCs has been well known to lower the immunological rejection and accelerate the revascularization after transplantation [11,37]. Of great interest in this context is our findings that co-culture of MCSs with PPCs has led to increased expression of angiogenic factors of MSCs, indicative of their intricate interaction. 

In fact, co-transplantation is an in vivo co-culture system. As evidenced by our co-transplantation experiments with the best glycemic control and glucose response, the direct interaction between MSCs and ICCs allowed the building of a symbiotic microenvironment and led to the increased function of ICCs in vivo. As discussed above, the MSCs are known to help speed up the process of vascularization and angiogenesis, which can attenuate the hypoxia and ischemia conditions after transplantation. Presumably, the revascularization effect is attributed to the angiogenic factors secreted by MSCs, including VEGF, TGF-β, etc. [41,42]. Moreover, the anti-inflammatory effects of MSCs have been widely reported, which decrease the tumor necrosis factor-alpha (TNF-α) level around pathological sites through a negative feedback mechanism of the release of soluble tumor necrosis factor receptor 1 (sTNFR1) [43]. Both the effects of angiogenesis and anti-inflammation are achieved by a number of soluble factors; apart from these, the direct MSCs–ICCs interaction may also benefit from the adhesion from cell extracellular matrix (ECM) and the cell–cell interactions. It is well-known that the ECM is vital to the development of stem cells and the change of ECM can direct the fate of co-cultured stem cells [44,45,46]. Thus, compared with the non-co-transplantation groups, the co-transplanted ICCs may enjoy the benefits from the interaction with ECM proteins secreted by MSCs. Since the 3D structure is believed to be very important to the function of ICCs, MSCs may also support the in vivo ICC function by creating adhesion junctions to maintain the ICC structures; it is achieved because of the strong ability of MSCs to create tight junctions in the direct cell–cell contact [47]. Taken together, the co-transplantation of MSCs/ICCs is a promising approach to serving as an alternative for islet transplantation in the future.

**Figure 9:**
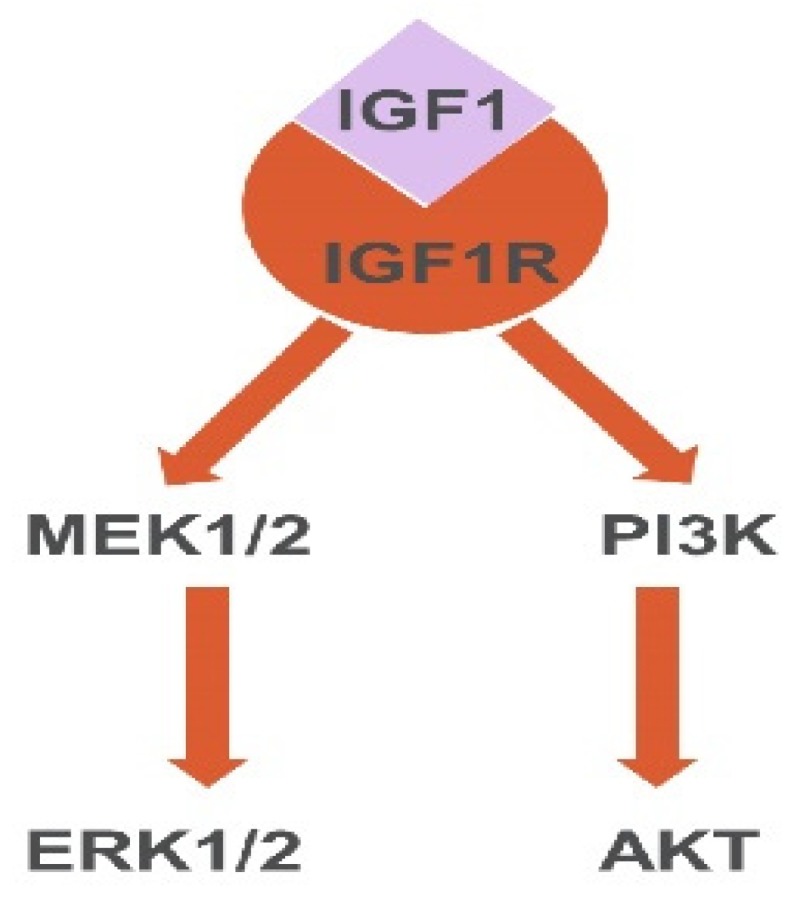
A schematic presentation summarizing the IGF1-IGF1R downstream pathways in PPCs. The activation of IGF1R by IGF1 can lead to the induction and further activation of Akt and ERK pathways.

On the other hand, our study findings further consolidate the notion for IGF1 being an indispensable player that participates in the beneficial effects of MSC and PPC interaction. It is well accepted that IGF1 signaling is implicated in the β-cell development during fetal and postnatal stages [48,49], which is also found to be involved in our MSC-induced PPC proliferation and differentiation. In this regard, the enhanced proliferation and differentiation were due to the activation of IGF1-IGF1R, as evidenced by the pharmacological inhibition data on PPP, an IGF1R inhibitor. In addition, it has been previously reported that two main pathways, PI3K and MAPK, which are closely related to cell proliferation, were found to be involved in beta cell development as well [50]. Moreover, these two pathways can be activated by various molecules including IGF1, yet the role of IGF1-PI3K/Akt or IGF1-MAPK in PPC proliferation has not been discussed before [51]. Indeed, our results revealed that PPP was able to attenuate Akt, PDK1, and ERK phosphorylation, subsequently reducing the proliferation stimulated by MSCs-CM. The nature of MSCs-CM is serum-free medium explaining why our results on the expression of the p-Akt and p-ERK in MSCs-CM groups did not display a higher level compared with those in the full serum condition. However, both the p-Akt and p-ERK levels were much higher compared with those in the serum-free and PPP + MSCs-CM groups, indicative of the effect of IGF1 on MSCs-CM in the activation of the phosphorylation of Akt and ERK. To be noted, the phosphorylation of PDK1 had even higher expression levels after treatment with MSCs-CM in relation to the full serum condition, suggestive of a stronger interaction between PDK1 and IGF1 or other trophic factors in MSCs-CM. Taken together, we posit that we have identified two downstream pathways of IGF1 in our culture system, namely Akt and ERK pathways, as shown in Figure 9. 

With the facilitation of the high-throughput method, the composition of the bone marrow-derived MSC-conditioned medium has been revealed including, but not limiting to, IGF1, VEGF, EGF, etc. [20]. Notably, IGF1 was reported to be at a very high expression level among those soluble factors [20], which is consistent with our data showing that the gene expression level of IGF1 in our co-cultured MSCs was increased significantly. Although we identified IGF1 as a key factor in our co-culture system, there should be other trophic factors that lend support for the development of PPCs, as demonstrated by the partially inhibitory effect of MSCs on PPC differentiation and proliferation after the blockade with IGF1R. 

VEGF is a well-known growth factor that can promote cell growth, for example, the bone morphogenetic proteins that enhance the propagation of endothelial cells via VEGF/VEGFR [52]. Despite the fact that VEGF can be secreted by MSCs, our PPCs were lacking the expression of VEGFR (data not shown), and thus the role of VEGF may rely on the MSC co-transplantation enhanced ICC function through the promotion of the angiogenesis, rather than the in vitro co-culture system. As for the EGF, it has already been put into use in our PPC expansion protocol and there is no doubt about the role of EGF in co-culture. Other factors such as the keratinocyte growth factor (KGF), angiotensin 2 (Ang 2), and transforming growth factor-β1 (TGFb1) may be also beneficial to the development of PPCs in all kinds of ways. In order to draw a fuller map or understanding of the mechanistic pathways for MSC-PPCs interaction-mediated beta-cell development, further investigations should be performed.

## 4. Materials and Methods

### 4.1. Human Ethics for the Use of Human Samples

Consent for using human fetal pancreas and bone marrow was approved by the Clinical Research Ethics Committee and agreed by donors from the Prince of Wales Hospital of the Chinese University of Hong Kong. Ethics approval of using human fetal tissues was obtained from the Joint Chinese University of Hong Kong-New Territories East Cluster Clinical Research Ethics Committee in September 2011 (CREC-2010.574 and CRE-2011.383).

### 4.2. Cell Culture of MSCs, PPCs, and ICCs

**Figure 10:**
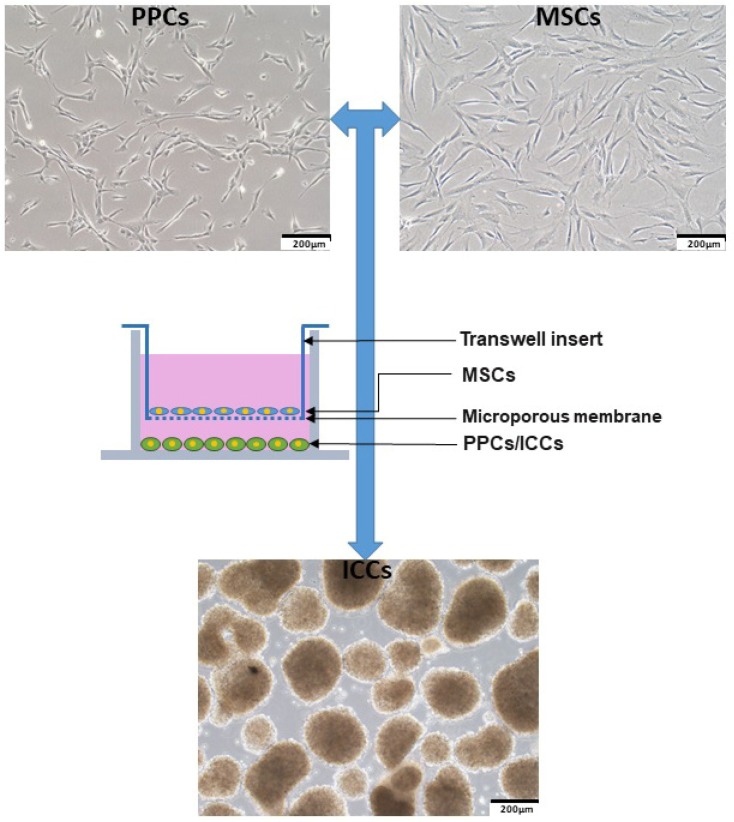
A schematic diagram depicting the set-up and composition of the co-culture system.

The preparation of human fetal PPCs/MSCs and differentiation of PPCs were procured as we described previously [4,53,54,55,56,57,58]. PPCs/MSCs from passage number under 10 were used in all the experiments. The cocktail for directed differentiation of PPCs was conducted as follows. PPCs were harvested by TrypLE Select (Invitrogen, Carlsbad, CA, USA) and cultured in an ultralow attachment plate (Corning, NY, USA) with differentiation cocktail medium-containing growth factors. The media were changed every other day for eight days to allow ICC formation. To observe the effects of MSCs on PPC differentiation, we established a co-culture system, as shown in Figure 10. MSCs were cultured in 1.0 μm inserts (Millipore, Bedford, MA, USA) overnight to achieve cell adherent. The medium was then changed into co-culture medium, which is an alpha-minimum essential medium (α-MEM, Manassas, VA, US) with 0.1% B27, 0.05% bovine serum albumin (BSA), without growth factors. Inserts with MSCs were finally hung over a 6-well ultralow attachment plate to allow PPCs to form 3D cell clusters. Medium was changed every two days for eight days to ensure ICC formation.

### 4.3. Assessment of PPC Proliferation and Apoptosis

To reveal the promotion of proliferation and differentiation on PPCs, MSC-conditioned medium and co-culture conditioned medium were harvested. Briefly, MSCs were cultured in a T-75 flask until they reached 80% confluence and were washed twice with phosphate-buffered saline (PBS) following the replacement of 12 ml serum-free α-MEM or co-culture medium for 48 h. Similarly, the PPC-conditioned medium for the study of angiogenic factors of MSCS was harvested with serum-free Roswell Park Memorial Institute (RPMI) medium.

For the PPC proliferation and cell death assay, PPCs were seeded in 96-well plates overnight and the medium was then replaced by serum-free α-MEM, full serum (cocktail) RPMI medium, or MSC-conditioned medium, respectively. In some studies, IGF1 (Invitrogen, Carlsbad, CA, USA) and PPP (Sigma-Aldrich St. Lois, MO, USA) were administrated into the culture system. PPCs were then cultured for another 48 h and cell proliferation was measured by BrdU (Boehringer Mannheim, Mannheim, Germany) while cell death was evaluated by Cell Death ELISA (Roche Diagnostics, Mannheim, Germany) according to the manufacturer’s protocol.

For the PPC cell cycle and apoptosis assay, PPCs were cultured in 6-well plates overnight and the medium was changed into serum-free, full serum (cocktail) RPMI medium, or MSC-conditioned medium, respectively. After 48 h, PPCs were harvested and a Cell Cycle and Apoptosis Analysis Kit (Beyotime, Shanghai, China) or FITC Annexin V Apoptosis Detection Kit I (BD Pharmingen, San Diego, CA, USA) was used before flow cytometry (LSRFortessa cell analyzer, BD Biosciences, Franklin Lakes, NJ, USA), according to the manufacturer’s protocol. Results were analyzed by BD FACSDiva software.

### 4.4. Analysis of mRNA Levels by Quantitative Real-Time PCR

Total RNA of the PPCs, MSCs, and ICCs were collected and subjected to reverse transcription. Resultant cDNAs were analyzed by a ViiA 7 Real-Time PCR system (Applied Biosystems Life Technologies, Austin, TX, USA). Gene expression levels normalized to β-actin were calculated by the comparative threshold cycle method (2^−ΔΔ^Ct). Human primer sequences are shown in Table 1.

### 4.5. Measurement of Insulin Content

Insulin of ICCs was extracted using acid ethanol (0.18 M HCl in 96% ethanol (vol/vol)) at 4 °C overnight as described previously [59], and the concentration was measured by an Ultra-sensitive Human Insulin Immunoassay Kit (The University of Hong Kong, Hong Kong, China) according to the manufacturer’s protocol.

### 4.6. Western Blot Analysis

Total protein was extracted by CytoBuster protein extraction reagent (Novagen, Madison, WI, USA) and fractionated by SDS/PAGE, and then was transferred to PVDF membranes (Bio-Rad, Chicago, IL, USA). Membranes were probed with primary antibodies including BAX, Bcl-2, Akt, p-Akt, ERK, p-ERK, PDK1, and p-PDK1 (Cell Signaling Technology, Beverly, MA, USA), normalized by β-actin (Santa Cruz Biotechnology, Santa Cruz, CA, USA)) or GAPDH (Cell Signaling Technology, Beverly, MA, USA).

### 4.7. Immunofluorescent Staining

PPCs were cultured in coverslips in a 6-well plate overnight. Medium was changed into serum-free, full serum, or MSC-conditioned medium in the presence or absence of IGF1 and PPP, respectively. PPCs were fixed using 4% paraformaldehyde for 8 minutes and then rinsed with cold PBS for 10 minutes twice. 

The fresh kidney and pancreas from sacrificed mice were removed and fixed with 4% paraformaldehyde (Sigma-Aldrich St. Lois, MO, USA) overnight. Then the kidney and pancreas were dehydrated with gradient sucrose (10%, 20%, and 30%). After being embedded by O.C.T (Sakura Finetek USA, Torrance, CA, USA), samples were frozen immediately by liquid nitrogen and stocked under -80 ℃. The cryotome (Thermo FSE, Cambridge, MA, USA) was used to cut the frozen embedded sample into 7 μm-thick cryostat sections and then allowed the sections to air dry at 25 °C.

The slides were then rinsed with cold PBS for 10 minutes twice. PBS containing 0.1% Triton X-100 was used to incubate slides for 4–10 minutes. Then the slides were blocked by 2% BSA/PBS for 60 minutes and probed with 1:250 Ki-67 antibody (Abcam, Cambridge, UK) 4 °C overnight. After being rinsed with PBS, fluorescent-conjugated secondary antibody (Invitrogen-Alexa, Carlsbad, CA, USA) was applied at 25 °C for 60 minutes followed with DAPI staining. After washing with PBS, slides were then mounted by mounting medium (Vector Laboratories, Burlingame, CA, USA). Insulin and Ki-67 positive cells were captured by Olympus FV1200 Inverted Confocal Microscope.

### 4.8. Transplantation of ICCs and Blood Glucose Homeostasis Measurement

The streptozocin (STZ) (Sigma-Aldrich St. Lois, MO, USA) was used to induce the T1DM animal model in nude mice (65 mg/kg for 5 days). The STZ-treated nude mice with blood glucose over 16.7 mM for at least one week were employed in the following studies. 1 × 10^6^ MSCs, cocktail, and co-culture derived ICCs (approximately 1000 ICCs) were transplanted, and 1 × 10^6^ MSCs + ICCs (approximately 1000 ICCs) were co-transplanted under renal capsule according to our reported protocols [4,60]. 

Body weight and blood glucose level were monitored for 6 weeks after the transplantation/co-transplantation. The pancreas and engrafted kidney were removed for immunohistochemical examination based on our previously described protocols [54,55,60,61].

The intraperitoneal glucose tolerance test (IPGTT) was performed 30 days post-transplantation according to our previously reported protocols [4]. In brief, all groups received the intraperitoneal injection of water dissolved glucose (1 g/kg body weight) after 6 h fasting, and the blood glucose was monitored at 0, 15, 30, 60, 90, and120 minutes after the injection of glucose.

### 4.9. Statistical Analysis

Data are expressed as means + SEM. Analysis was conducted in GraphPad Prism. One-way analysis of variance followed by Tukey’ or Student’s *t* test were applied for comparison. P < 0.05 was considered statistically significant.

## 5. Conclusions

In conclusion, our data indicate that MSCs stimulate the differentiation and proliferation of human PPCs via the mediation of IGF1 and its downstream signaling pathways of Akt and ERK. The co-transplantation of MSCs and ICCs significantly enhanced the functionality of ICCs. It is plausible to propose that MSC co-transplantation/co-culture with ICCs can greatly improve both the in vitro and in vivo function of PPCs/ICCs. This finding sheds new light into a fuller understanding of the interaction between PPCs and MSCs and, more importantly, a feasible and economical platform for the clinical practice of islet transplantation.

## Figures and Tables

**Table 1:** Primer Sequences.

Gene Name	Forward (5′-3′)	Reverse (5′-3′)	
VEGF	CTACCTCCACCATGCCAAGT	GCAGTAGCTGCGCTGATAGA	
TGF-β	CCCAGCATCTGCAAAGCTC	GTCAATGTACAGCTGCCGCA	
IL-6	GGTACATCCTCGACGGCATCT	GTGCCTCTTTGCTGCTTTCAC	
INSULIN	CAGCCTTTGTGAACCAACACC	GGTCTTGGGTGTGTAGAAGAAGC	
IGF1	TGGATGCTCTTCAGTTCGTG	TGGTAGATGGGGGCTGATAC	
IGF1R	GCACCATCTTCAAGGGCAATTTG	AGGAAGGACAAGGAGACCAAGG	
NGN3	CGGACCCCATTCTCTCTTCT	ACTTCGTCTTCCGAGGCTCT	
NEUROD	TCCAAAATCGAGACTCTGCGC	GCAAAGCGTCTGAACGAAGGA	
NKX2.2	TCTCCTTGGAGTGGCAGATTC	AAACACGGCGTAGAGTTCAGC	
NKX6.1	GACGGGAAGAGAAAACACACG	ACTCTCTGTCATCCCCAACGA	
PDX1	ACTCCACCTTGGGACCTGTTT	TTAAGGTACTCGGCCCAGCTT	
ACTIN	TGTCCACCTTCCAGCAGATGT	CGGACTCGTCATACTCCTGCTT	
ISL-1	GATCAAATGCGCCAAGTGCAG	CAGCGGAAACACTCGATGTGA	
MIP-2	CGCCCAAACCGAAGTCAT	GATTTGCCATTTTTCAGCATCTTT	

## Abbreviations

PPCs	Pancreatic progenitor cells
ICCs	Islet-like cell clusters
MSCs	Mesenchymal stem cells
IGF1	Insulin-like growth factor 1
IGF1R	Insulin-like growth factor 1 receptor
DM	Diabetes mellitus
T1DM	Type 1 diabetes mellitus
T2DM	Type 2 diabetes mellitus
MSCs-CM	MSC-conditioned medium
PPCs-CM	PPC-conditioned medium
PPP	Picropodophyllin
α-MEM	Alpha-minimum essential medium
RPMI	Roswell Park Memorial Institute
BSA	Bovine serum albumin
FBS	Fetal bovine serum
PCR	Polymerase chain reaction
PBS	Phosphate-buffered saline
PI3K	Phosphoinositide 3-kinases
MAPK	Mitogen-activated protein kinase
Akt	Protein kinase B
ERK	Extracellular signal-regulated kinases
MEK	Mitogen-activated protein kinase
p-	phospho
Bcl-2	B-cell lymphoma 2
BAX	BCL2 Associated X
PDK1	Phosphoinositide-dependent kinase-1
VEGF	Vascular endothelial growth factor
VEGFR	Vascular endothelial growth factor receptor
TGF	Transforming growth factor
TGF-β	Transforming growth factor-β
IL-6	Interleukin-6
NGN3	Neurogenin-3
NEUROD	Neurogenic differentiation
NKX2.2	NK2 homeobox 2
NKX6.1	NK6 homeobox 1
PDX1	Pancreas and duodenal homeobox gene 1
ISL-1	ISL LIM homeobox 1
GAPDH	Glyceraldehyde 3-phosphate dehydrogenase
MIP-2	Macrophage inflammatory protein 2
IPGTT	Intraperitoneal glucose tolerance test
STZ	Streptozocin
EGF	Epidermal growth factor
HGF	Hepatocyte growth factor
KGF	Keratinocyte growth factor
Ang 2	Angiotensin 2
sTNFR1	Soluble tumor necrosis factor receptor 1
ECM	Extracellular matrix
TNF-α	Tumor necrosis factor-alpha
ESCs	Embryonic stem cells
